# Increase of gap junction activities in SW480 human colorectal cancer cells

**DOI:** 10.1186/1471-2407-14-502

**Published:** 2014-07-09

**Authors:** Kristina Bigelow, Thu A Nguyen

**Affiliations:** 1 Department of Diagnostic Medicine/Pathobiology, Kansas State University, 1800 Denison Ave., Manhattan, KS 66506, USA

**Keywords:** Gap junction intercellular communication, PQ1, Kinase activity

## Abstract

**Background:**

Colorectal cancer is one of the most common cancers in the United States with an early detection rate of only 39%. Colorectal cancer cells along with other cancer cells exhibit many deficiencies in cell-to-cell communication, particularly gap junctional intercellular communication (GJIC). GJIC has been reported to diminish as cancer cells progress. Gap junctions are intercellular channels composed of connexin proteins, which mediate the direct passage of small molecules from one cell to the next. They are involved in the regulation of the cell cycle, cell differentiation, and cell signaling. Since the regulation of gap junctions is lost in colorectal cancer cells, the goal of this study is to determine the effect of GJIC restoration in colorectal cancer cells.

**Methods:**

Gap Junction Activity Assay and protein analysis were performed to evaluate the effects of overexpression of connexin 43 (Cx43) and treatment of PQ1, a small molecule, on GJIC.

**Results:**

Overexpression of Cx43 in SW480 colorectal cancer cells causes a 6-fold increase of gap junction activity compared to control. This suggests that overexpressing Cx43 can restore GJIC. Furthermore, small molecule like PQ1 directly targeting gap junction channel was used to increase GJIC. Gap junction enhancers, PQ1, at 200 nM showed a 4-fold increase of gap junction activity in SW480 cells. A shift from the P0 to the P2 isoform of Cx43 was seen after 1 hour treatment with 200 nM PQ1.

**Conclusion:**

Overexpression of Cx43 and treatment of PQ1 can directly increase gap junction activity. The findings provide an important implication in which restoration of gap junction activity can be targeted for drug development.

## Background

Colorectal cancer is the third most common cancer and the third leading cause of cancer related death in the United States [1,2]. In 2013, approximately 136,830 people were diagnosed with colorectal cancer. Approximately 50,310 deaths in the past year were due to colorectal cancer [3]. Thus, understanding the etiology of colorectal cancer is critical for the treatment of the disease.

GJIC has been shown to be decreased in cancerous cells and at tumor borders [4,5]. Gap junctions are intercellular channels made of the protein known as connexin. There are 21 isoforms of connexin [6]. Six connexins make up a connexon; two connexons, each on an adjacent cell, interact and form a gap junction. Gap junctions mediate the direct passage of small molecules (<1000 Da) from one cell to the next [7]. They are involved in the regulation of the cell cycle, cell differentiation, and cell signaling [8]. The life cycle of gap junctions is regulated by phosphorylation events [9-11].

Regulation of Cx43 in GJ formation has been shown to be due to phosphorylation by different kinases at multiple phosphorylation sites on the carboxy(C)-terminus domain [9-14]. Mitogen-activated protein kinase (MAPK) and active protein kinase B (pAKT) are known regulators of Cx43 [12-14]. Early studies showed MAPK phosphorylation of Cx43 which leads to a decrease in GJs. However, recent literature suggests that it can lead to an upregulation in Cx43 causing an increase in functional GJs [13]. Another kinase, Akt, has been shown to stabilize gap junctions via phosphorylation, the exact site of Akt phosphorylation Cx43 is unknown. Dunn et al., found that upon inhibition of Akt by Akt VIII inhibitor or with a dominate negative version of Akt gap junctions were smaller and less phosphorylated Cx43 was present [15].

Phosphorylation events correlate with three known isoforms of Cx43, the isoforms are known as P0, P1 and P2. The P0 form localizes on internal membranes like the Golgi apparatus and the endolysosomal system [16,17]. The P1 and P2 forms are associated with certain phosphorylation cites. The P1 form has phosphorylation site at S364/S365. When phosphorylated at S365 of Cx43, the assembly conformation of the gap junction was observed [18]. The P2 form has two different sets of phosphorylation sites. One of these sets is phosphorylated at S325/S328/S330; this form has been found at the stage of gap junctional plaques. When phosphorylated at S262 and/or S368 of Cx43, a decrease in GJIC is found [17,19,20].

Gap junction enhancers, 6-Methoxy-8-[(3-amionpropyl) amino]-4-methyl-5-(3-trifluoromethyl-phenloxy) quinolone (PQ1), has been demonstrated to increase gap junction activity in breast cancer cells [21]. PQ1 caused an 8.5-fold increase in gap junction activity in T47D breast cancer cells and subsequently a decrease of 70% growth in a xenograft tumor [21]. Furthermore, PQ1 has also been shown to induce apoptosis via caspase 8 and 9 [22]. Oral bioavailability studies indicate that administration of PQ1 via oral gavage has a low toxicity to normal tissue with no observable adverse effects [23], while significantly attenuating tumor growth [21].

As colorectal cancer forms, there is a decrease in gap junction activity and Cx43 expression as well as a shift in localization of Cx43 [4,5,24]. Thus, this study addresses whether overexpression of Cx43 or increase gap junction activity can be achieved in human colorectal cancer cells, SW480. Using overexpression of Cx43 and treatment of PQ1 approaches, the gap junction activity of SW480 cells was restored. Overall, this study provides evidence for the first time that regain of GJIC can be achieved by a small molecule gap junction enhancer, PQ1, on SW480 colorectal cancer cells.

## Methods

### Ethics statement

All experiments in this manuscript have been approved by the Kansas State University Institutional Biosafety Committee (IBC).

### Cell Line

The SW480 human colorectal cancer cell line was purchased from American Type Cell Culture (ATCC, Manassas, VA). Cells were grown with 0% CO_2_ in Leibovitz’s L-15 Medium with 10% Gibco Fetal Bovine Serum (FBS) purchased from Life Technologies (Grand Island, NY, USA).

### Western blot

Cells were seeded to 50% density in a T-25 cm^2^ flask for 24 hours and allow the density to reach 90% prior to treatment. cells were harvested with lysis buffer (20 mM Tris–HCl pH 7.6, 0.5 mM EDTA, 0.5 mM EGTA, and 0.5% Triton-X 100) (Cell Signaling Technology Inc., Danver, Massachusetts, USA). The mixture was centrifuged at 13,000 rpm (15,700 g using an Eppendorf centrifuge 5415R with rotor F-45-24-11, Eppendorf North America, Hauppauge, New York, USA) for 30 minutes at 4˚C, and the supernatant was collected. Total protein concentration was determined using a Bio-Rad protein assay kit (Bio-Rad Life Science Research, Hercules, California, USA). 25 μg of whole cell extract was separated by 5-10% sodium dodecylsulfate (SDS) polyacrylamide gel electrophoresis (PAGE) and transferred onto a nitrocellulose membrane. The nitrocellulose membrane was immunoblotted against the protein of interest. The primary antibodies were purchased from two different companies; mouse anti-Cx43 antibody and the mouse anti-GAPDH antibody were purchased from Santa Cruz Biotechnology (Santa Cruz, California, USA). Primary antibodies purchased from Cell Signaling Techonolgy (Danvers, Massachusetts, USA) were; rabbit anti-phospho-Akt and rabbit anti-phospho-p44/42 MAPK. Secondary antibodies were anti-mouse and anti-rabbit IGg HRP linked, they were purchased from Cell Signaling Technology (Danver, Massachusetts, USA). Proteins were detected using the FluorChem E System purchased from Protein Simple (Santa Clara, California, USA).

### Transfection

Eight hundred thousand SW480 cells were seeded into six-well plates for 24 hours. Cells were transfected with 3.5 μg of Gja1, NM 012567.2, subcloned into pEGFP-N3 vector [25] and Optifect reagent in 0% FBS tissue culture media.

### Gap junction activity

Scrape Load/Dye Transfer (SL/DT) assay was used to measure gap junction activity. Eight hundred thousand cells were grown on a cover slip in a six-well plate. Cells were grown for 24 hours; cells designated for overexpression of Cx43 were transfected, 24 hours later treatments of 200 nM 12-O-Tetradecanoylphorbol-13-Acetate (TPA) and/or with 50 nM, 200 nM and 500 nM PQ1 for 1 hour. Cells were then washed with Phosphate Buffered Saline (PBS) 3 times. A mixture of 1% Lucifer yellow and 0.75% Rhodamine dextran was added in the center of the cover slip. Two cuts crossing one another in the center of the coverslip were made. After 3 minutes, cells were washed with PBS 3 times and incubated at 37˚C in tissue culture media for 20 minutes. The cells were then washed with PBS and fixed with 2.5% paraformaldehyde for 30 minutes. Cells were mounted on a slide and then sealed and visualized under a fluorescent microscope (Nikon Eclipse 80i, Nikon Instruments, Melville, NY, USA) (X-Cite 120 PC fluorescence illumination system, EXFO Photonic Solutions Inc., Mississauga, Ontario, Canada) at 10x objective (Nikon Instruments, Melville, NY, USA). Images were captured using Nikon Digital Sight Fi1 (Nikon Instrument, Melville, NY, USA). The distance between the designated cut and the dye transfer was measured. The distance of dye uptake indicates that cells are active and have allowed the dye to pass from one cell to the next cell.

### Proliferation and viability

Eight hundred thousand cells seeded into 6-well plates for 24 hours. Cells were treated with PQ1 at various concentrations or overexpressed with Cx43. After another 24, 48 or 72 hours tissue culture media of respective treatments was saved and 0.5 mL of trypsin was added to the cells for 5 minutes. Three mL of PBS was used to harvest cells. Cells were spun down for 5 minutes at 13,000 rpm; afterwards solution of tissue culture media, trypsin and PBS was removed. Nine hundred μL of PBS and 100 μL of trypan blue were added to the pellet and left to stand for 5 minutes. Cellometer Auto 2000 from Nexcelom Bioscience was used to measure number of cells for proliferation and viability.

### Statistical analysis

Pixel intensities of protein bands were normalized to pixel intensities of loading control (GAPDH). All protein expression data were expressed as mean ± standard deviation of three independent experiments. Significant differences were analyzed by comparing the data of treated samples and control (untreated) samples and indicated as *P* value > 0.05 using Student’s *t*-test.

## Results

### Transfection of Cx43 leads to increased GJIC in SW480 colorectal cancer cells

**Figure 1:**
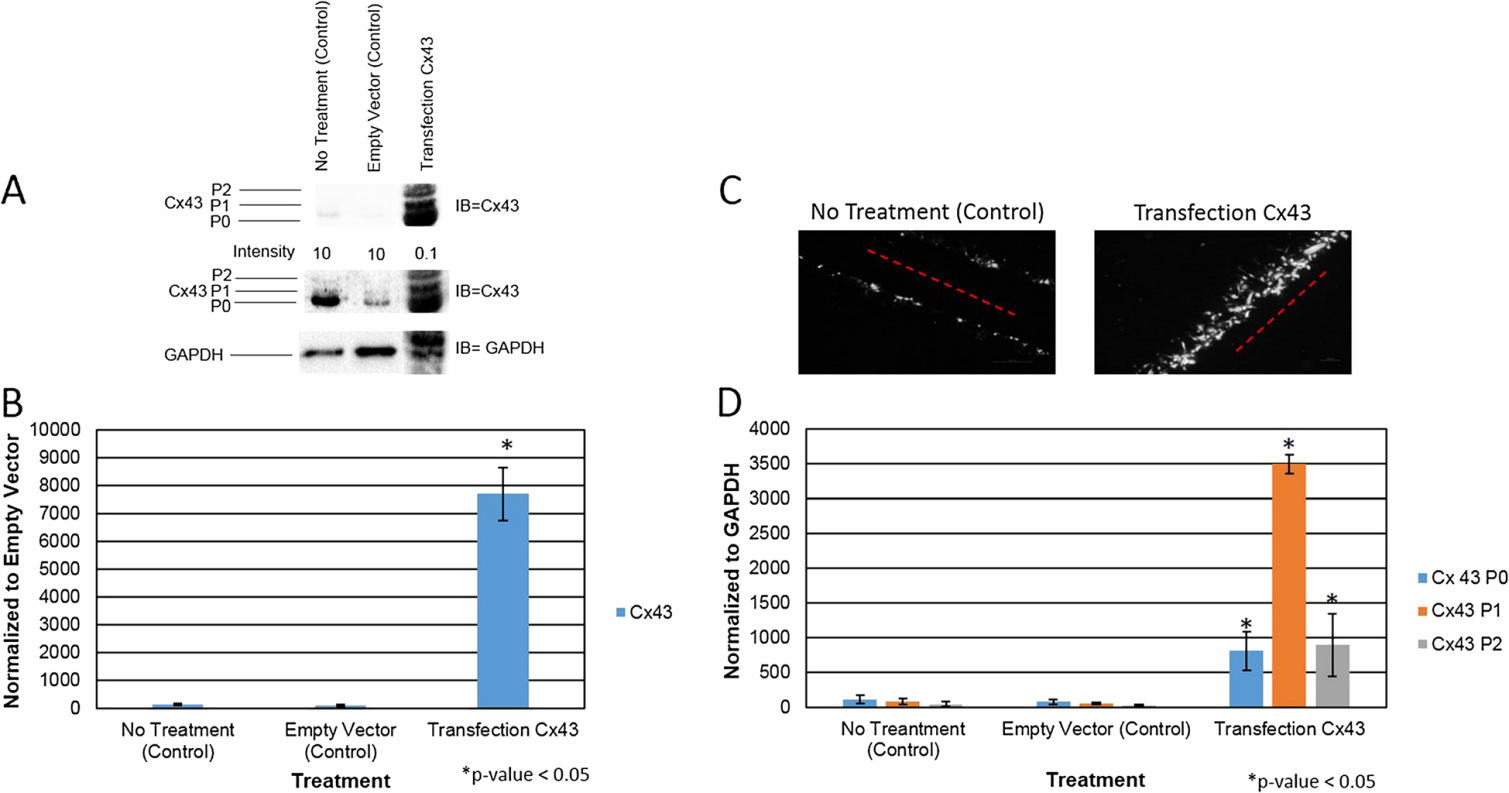
**Overexpression of Cx43 increases gap junction activity.** Cells were treated with: no transfection (control), transfection of Empty Vector (control), transfection of Cx43 for 24 hours. Level of Cx43 and its isoforms were examined by western blot analysis. GAPDH was used as a loading control. **A)** Levels of Cx43 were examined using anti-connexin43 (F-7) antibody specific for amino acids 357–381 at the C-terminus domain. **B)** Graphical presentation of three independent experiments showing pixel intensities of total Cx43 normalized to Empty Vector (control). **C)** Scrape Load/Dye transfer assay (SL/DT) was performed after no transfection and the transfection of overexpression of Cx43. Lucifer yellow dyes in cells indicate in white. Red line indicates the point of entry for Lucifer yellow. **D)** Graphical presentation shows the ratio of Cx43 isoforms P0, P1 and P2 from panel **A**. Data were obtained in three independent experiments and are represented as the mean ± SD. *P value is <0.05 compared to control. IB = Immunoblot against Cx43.

**Figure 2:**
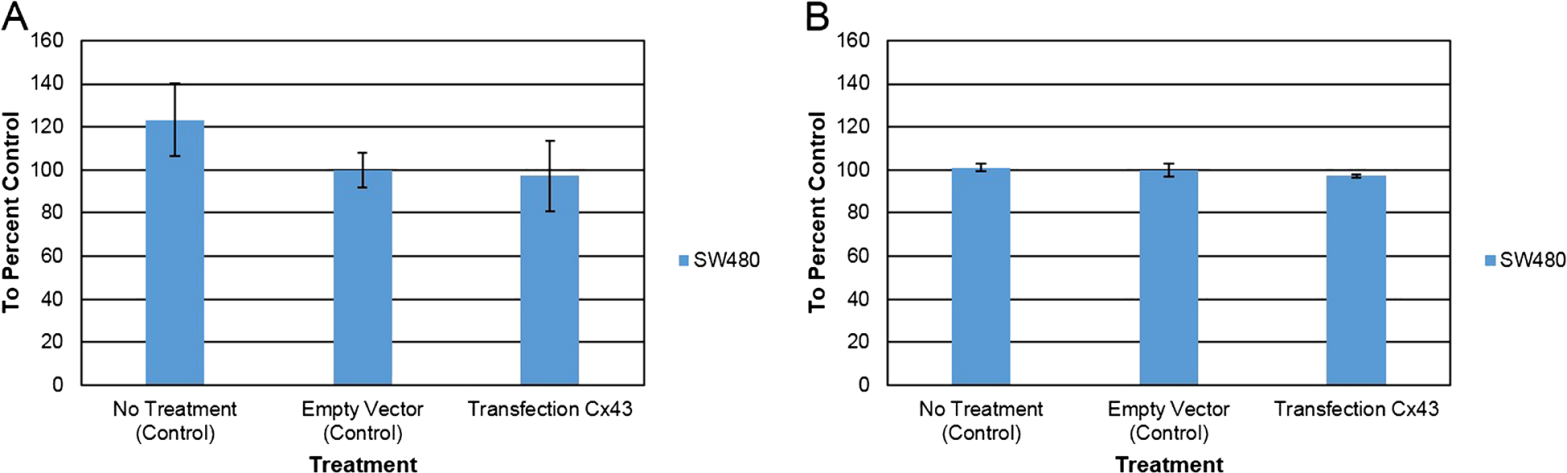
**Overexpression of Cx43 causes a decrease in viability of SW480 cells.** Eight hundred thousand cells were seeded into six-well plates. Cells were transfected with optifect for 24 hours. Transfection conditions were: Control, Empty Vector, and Cx43 vector. After 24 hours, viability and proliferation assays were performed. **A)** Proliferation of SW480 cells. **B)** Viability of SW480 cells. Data were obtained in three independent experiments and are represented as the mean ± SD.

Intercellular communication in many organs is maintained via GJIC. An effective clinical drug targeting GJIC has not been studied for colorectal cancer at this time; thus, ways to increase GJIC in colorectal cancer cells were examined. Cells were transfected with Cx43 expression plasmid for 24 hours. Western blot analysis shows that 25 ug of Cx43 expression vector was sufficient to increase Cx43 in SW480 cells compared to control or empty vector (Figure 1A and B). These cells were analyzed for gap junction activity after 24 hours of transfection. The results showed a 6-fold increase of gap junction activity in Cx43-transfected cells compared to control cells (Figure 1C). Thus, these suggest that regain of GJIC in SW480 cells can be achieved via transfection of Cx43. Furthermore, differential pattern of Cx43 isoform was observed. The protein blot analysis shows that there are three distinct isoforms of Cx43: P0, P1, and P2. Isoform expression of Cx43 has shifted from P0 form to P1 form in the Cx43 transfected cells (Figure 1D). Overall these results show an increase in GJIC by overexpression of Cx43.The effects of overexpression of Cx43 on cell viability and proliferation were analyzed. Proliferation study of SW480 cells, overexpressed with or without the Cx43 expression vector, show a decrease of 20% compared to control (Figure 2A). Viability of SW480 cells overexpressed by Cx43 was found to decrease by 4% (Figure 2B). These data demonstrate that the transfection did not alter the proliferation and viability of SW480 cells and the change in gap junction activity is due to the overexpression of Cx43.

### PQ1, gap junction enhancer, increases GJIC in SW480 colorectal cancer cells

**Figure 3:**
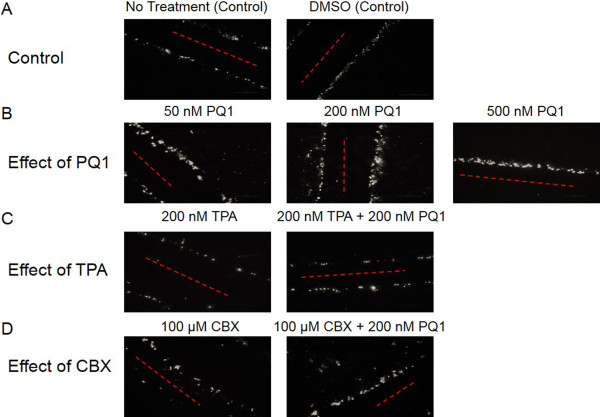
**PQ1 causes an increase in gap junction activity of SW480 cells.** Eight hundred thousand cells were seeded into six-well plates. SL/DT assay was performed after the following conditions: **A)** SW480 cells were treated with no treatment (control) and DMSO (control) for 1 hour; **B)** SW480 cells were treated with 50 nM PQ1, 200 nM PQ1 and 500 nM PQ1 for 1 hour; **C)** SW480 cells were treated with 200 nM TPA and 200 nM TPA + 200 nM PQ1 for 1 hour; and **D)** SW480 cells were treated with 100 μM CBX and 100 μM CBX + 200 nM PQ1 for 1 hour. Cells with Lucifer yellow indicated in white and the point of entry for Lucifer yellow indicated by red line. These images represent one set of three independent experiments.

The approach of increasing GJIC directly has potential to enhance the efficacy of cancer treatment. Since transfecting all cancer cells with Cx43 is not valid as a therapeutic option, an alternate approach is needed. Recently, gap junction enhancers, a class of substituted quinolines (PQs), have shown to increase GJIC in other cancer cells. Thus, in this study PQ1 was used to increase GJIC in SW480 colorectal cancer cells. SW480 cells were treated with PQ1 at concentrations of 50 nM, 200 nM and 500 nM for 1 hour. The gap junction activity was measured by scrape load/dye transfer assay. The results show that cells treated with 200 nM PQ1 have a 4-fold increase of dye transfer compared to control cells without treatment or solvent alone (Figure 3A and B). Interestingly, cells treated with PQ1 and GJ inhibitor, TPA, have no increase of gap junction activity compared to control, suggesting that TPA blocks PQ1-mediated GJIC in SW480 cells (Figure 3C). Carbenoxolone (CBX) is a known gap junction inhibitor; thus, it was used as a control to see if PQ1’s effects directly affect the gap junction. CBX is thought to effect the gap junctions by altering local lipid environment [26]. The effects of CBX on the bilipid layer membrane conductance were not studied in this paper. Results show that treatment with 100 μM CBX + 200 nM PQ1 did not show an increase in GJIC when compared to 100 μM CBX alone or controls (Figure 3A and D). This concludes that PQ1 cannot open GJs in the presence of a GJ inhibitor.

**Figure 4:**
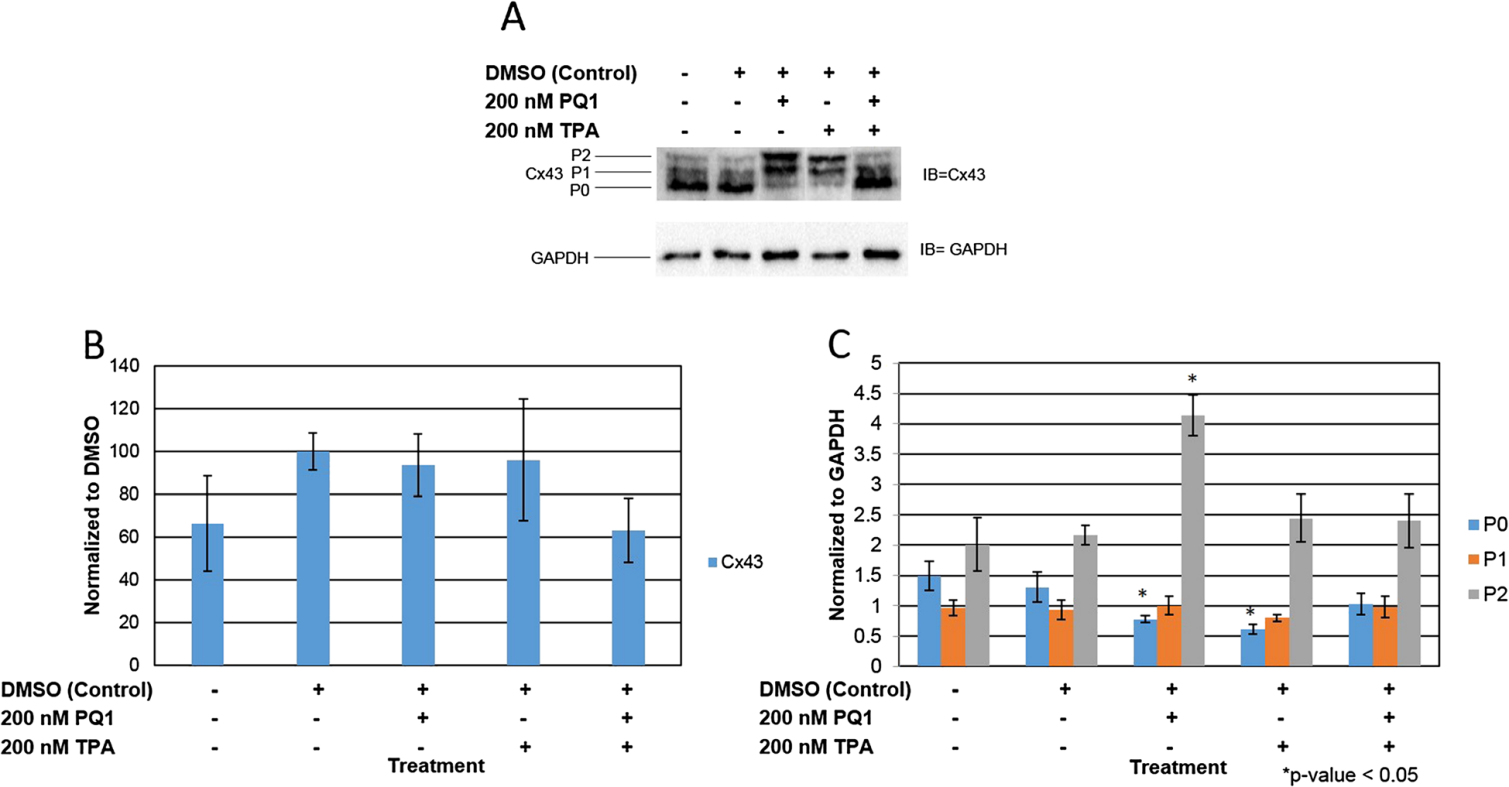
**PQ1 changes isoform expression of Cx43.** Cells were treated with: no treatment (control), DMSO (control), 200 nM PQ1, 200 nM TPA and 200 nM PQ1 + 200 nM TPA for 1 hour. Level of Cx43 and its isoforms were examined by western blot analysis. GAPDH was used as a loading control. **A)** Levels of Cx43 were detected using anti-connexin43 (F-7) antibody specific for amino acids 357–381 at the C-terminus domain. **B)** Graphical presentation of three independent experiments showing pixel intensities of total Cx43 normalized to control. **C)** Graphical presentation shows the ratio of Cx43 isoforms P0, P1 and P2. Data were obtained in three independent experiments and are represented as the mean ± SD. *P value is <0.05 compared to control. IB = Immunoblot against Cx43.

**Figure 5:**
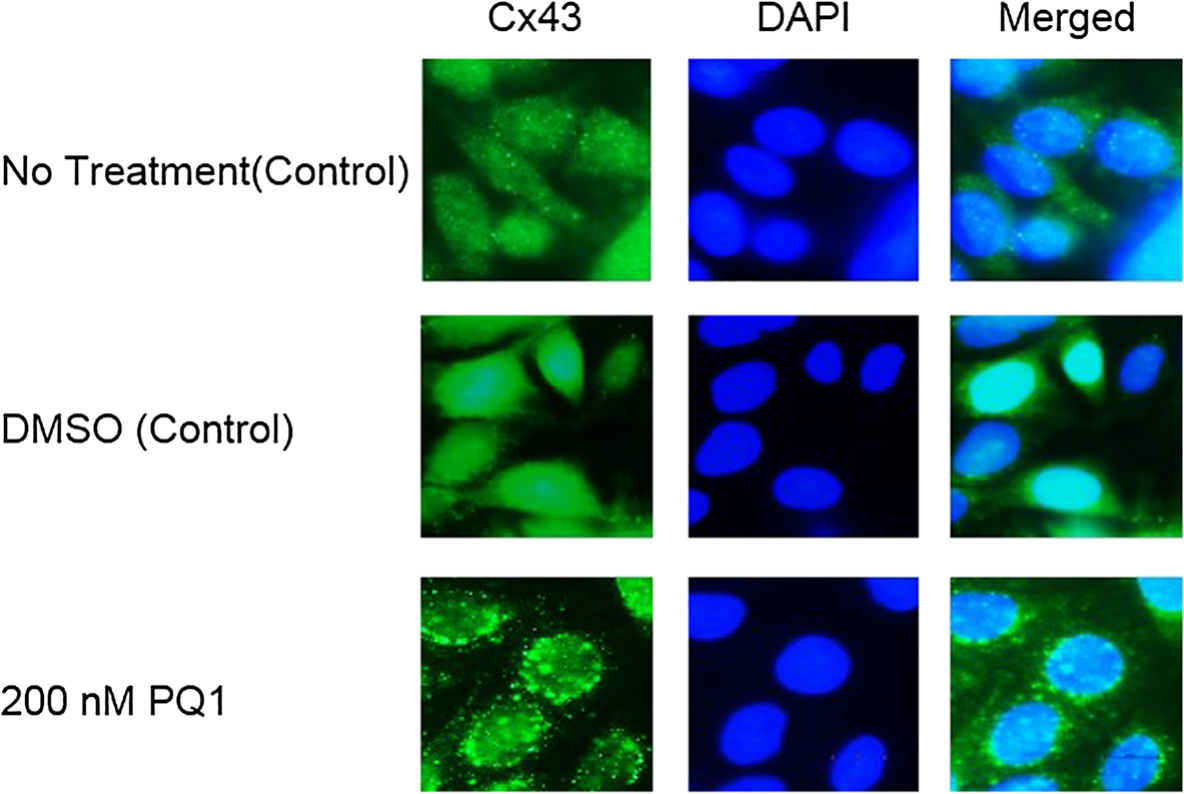
**Immunofluorescence of SW480 cells.** Eight hundred thousand cells were seeded into six-well plates. SW480 cells were treated with no treatment (control), DMSO (control) and 200 nM PQ1. Immunofluorescence was performed after 1 hour of PQ1 treatment.

Previously, PQ1 was constructed using the structure of the c-terminus of Cx43 [21]. Western blot analysis was used to analyze the effects of PQ1 on gap junction protein Cx43. Figure 4B shows the protein expression of Cx43 after treating with 200 nM PQ1 and/or 200 nM TPA for 1 hour. Results show no significant change in total Cx43 expression after treatment with PQ1 for 1 hour (Figure 4A and B). However, treatment with 200 nM TPA for 1 hour decreases expression of Cx43 by 30% (Figure 4A and B). These data are consistent with the findings by Oh et al., demonstrating that TPA leads to a decrease in Cx43 expression [27]. Figure 4C shows Cx43 isoform expression in treated cells compared to control cells. After treatment for 1 hour with 200 nM PQ1 a shift in isoform expression was seen from P0 in control cells to P2 as the dominant form in PQ1 treated SW480 cells (Figure 4A and C). The expression of the P2 form increased 2-fold after treatment with 200 nM PQ1 for 1 hour (Figure 4A and C). The P0 form decreased 2-fold and the P1 form had no change after PQ1 treatment. When treating cells for 1 hour with 200 nM TPA, the isoform profile of Cx43 shows no significant change in the expression of the P2 or P1 isoforms of Cx43 compared to control. The P0 isoform was seen to decrease 2-fold with TPA treatment alone when compared to the control P0 isoform. However, there was no significant change in isoform expression after treatment with both TPA and PQ1 for 1 hour, suggesting a potential antagonistic relationship between PQ1 and TPA. Immunofluorescence was used to visualize Cx43 localization after 200 nM PQ1 treatment for 1 hour compared to no treatment and DMSO controls. Results show a shift from Cx43 localization in the cytoplasm to Cx43 gap junctional plaques after PQ1 treatment (Figure 5). Overall, these findings suggest that PQ1’s ability to increase GJIC is by acting on the existing Cx43 and not by increasing Cx43 expression.

### PQ1’s effects on Kinase activity

**Figure 6:**
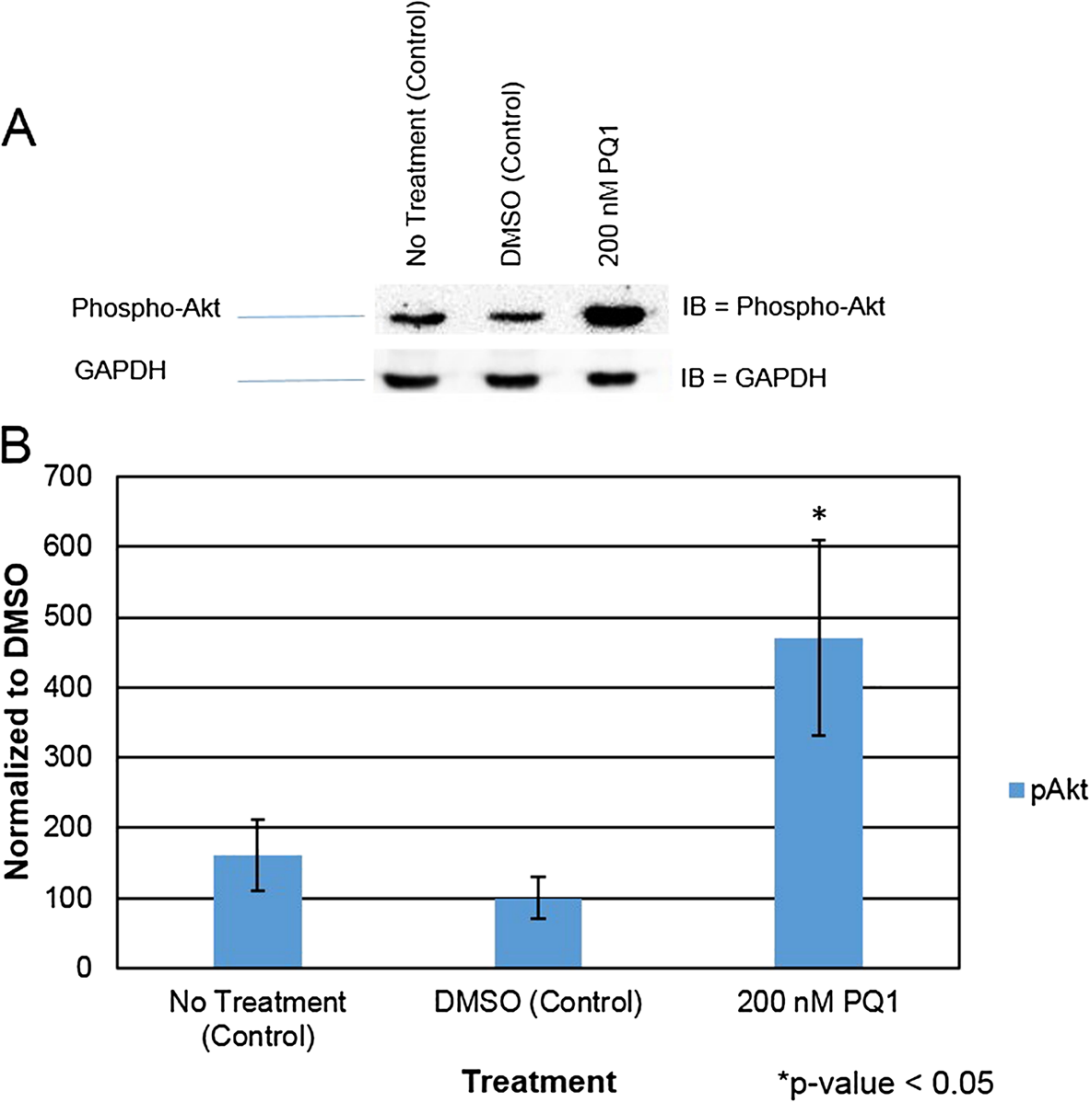
**PQ1 causes activation of Akt.** Cells were treated with: no treatment (control), DMSO (control) and 200 nM PQ1 for 1 hour. Level of phospho-Akt (active Akt) was examined by western blot analysis. GAPDH was used as a loading control. **A)** Level of active Akt was detected using anti-phospho-Akt (Ser473) (D9E) antibody specific for activated Akt. **B)** Graphical presentation of three independent experiments showing pixel intensities of active Akt normalized to control. *P value is <0.05 compared to control. IB = Immunoblot against phospho-Akt.

**Figure 7:**
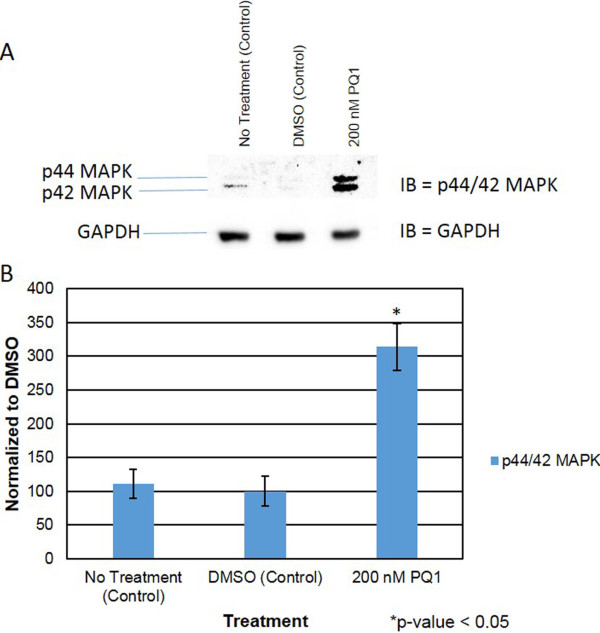
**PQ1 leads to p44/42 MAPK expression.** Cells were treated with: no treatment (control), DMSO (control) and 200 nM PQ1 for 1 hour. Levels of phospho-p44/42 MAPK (active p44/42 MAPK) expression was examined by western blot analysis. GAPDH was used as a loading control. **A)** Level of active p44/42 MAPK was detected using anti-phospho-p44/42 MAPK (Erk1/2) (Thr202/Tyr204) antibody specific for endogenous active p44/42 MAPK. **B)** Graphical presentation of three independent experiments showing pixel intensities of active p44/42 MAPK normalized to control. *P value is <0.05 compared to control. IB = Immunoblot against phosphor-p44/42 MAPK.

**Figure 8:**
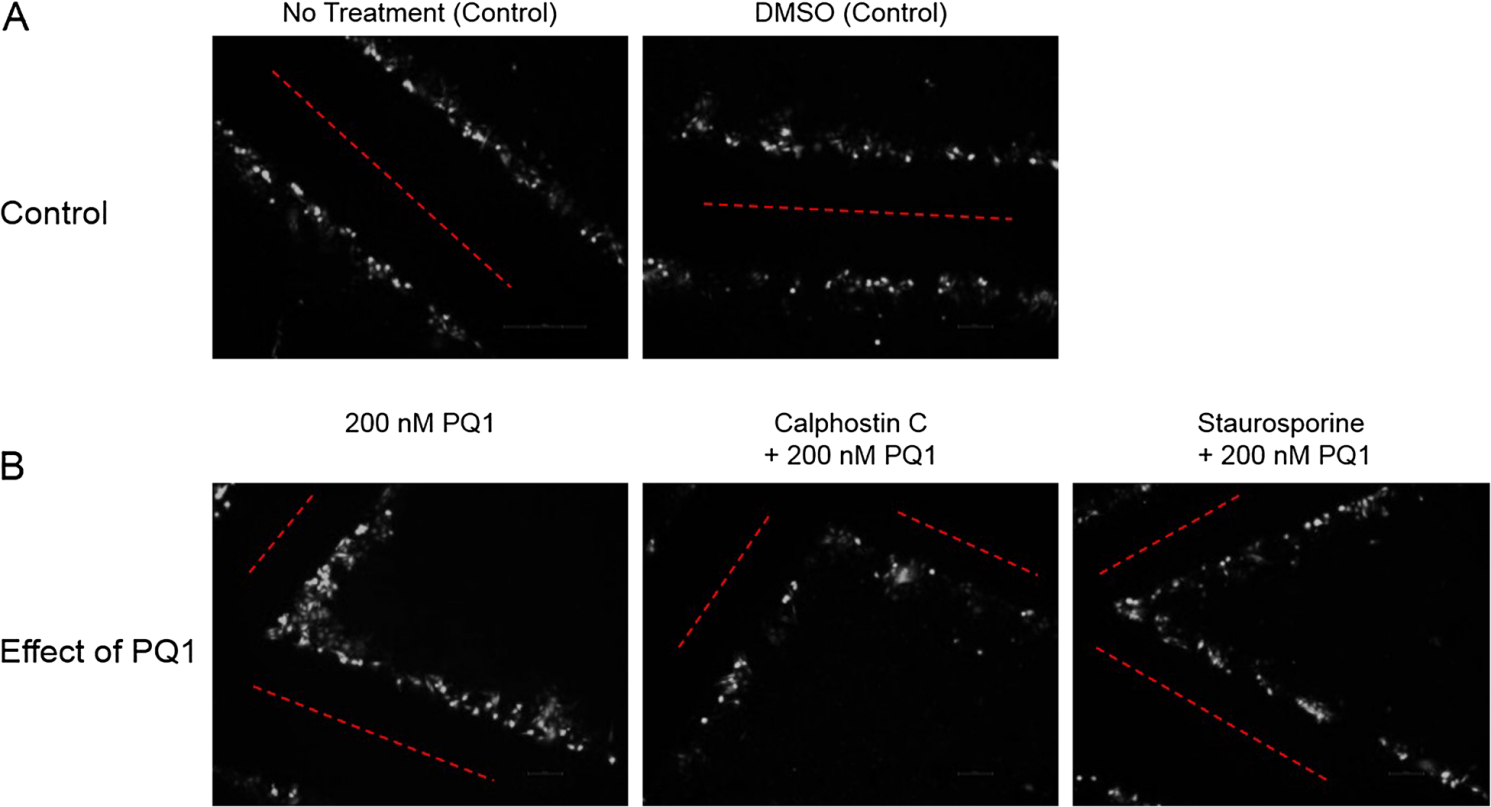
**PQ1’s effects on GJIC in the presence of kinase inhibitors.** Eight hundred thousand cells were seeded into six-well plates. **A)** SW480 cells were treated with no treatment (control) and DMSO (control) for 1 hour. **B)** SW480 cells were pre-treated with kinase inhibitors (Calphostin C or Staurosporin) for 1 hour. After pre-treatment, cells were treated with 200 nM PQ1 for 1 hour. SL/DT was preformed after 1 hour of PQ1 treatment. Cells with Lucifer yellow indicate in white and the point of entry for Lucifer yellow indicates in red line. These images represent one set of three independent experiments.

Since specific kinases phosphorylate Cx43 and lead the change in gap junction activity, this study focuses on key kinases involving the PQ1-mediated GJIC. The activated form of Akt and p44/42 MAPK were analyzed. After 1 hour of treatment with PQ1, activated Akt increases by 100% compared to control (Figure 6). This suggests that increasing gap junction activity by PQ1 may involve the activation of Akt.Furthermore, MAPK was examined due to the fact that MAPK can phosphorylate Cx43 and subsequently increase gap junction activity. The results show that an increase of 250% in activated form of MAPK (p44/42) was detected in the presence of 200 nM PQ1 at 1 hour treatment compared to controls (Figure 7). This provides the initial evidence that PQ1 may also involve in the activation of p44/42 MAPK and subsequently increase GJIC in SW480 cells.As PQ1’s activation of Akt and p44/42 MAPK may be independent of gap junctions, cells were pre-treated with kinase inhibitors calphostin C and staurosporin prior to treatment with 200 nM PQ1 for 1 hour. Figure 8 shows that in the presence of kinase inhibitors PQ1 does not cause an increase in GJIC compared to PQ1 treatment alone. This suggests that PQ1 leads to an increase in GJIC via kinases. The inhibitors used are specific to PKC so in the future inhibitors for MAPK and Akt will be used to test PQ1’s ability to increase GJIC without MAPK and Akt.

## Discussion

In colorectal cancer, a decrease in GJIC has been found. In this study, overexpression of Cx43 increased GJIC through the increase of gap junction protein, Cx43 (Figure 1). It was shown that overexpression of Cx43 lead to a change in isoform expression from P0 to P1 leading to an increase in GJIC. This suggests that the SW480 cells have the key kinases needed to regulate GJIC. Other approach of increasing GJIC was also evaluated since transfection of Cx43 is not a viable therapeutic target. A small molecule, PQ1, was tested as a potential GJ enhancer in SW480 colorectal cancer cells. The results show that PQ1 can increase gap junction activities.

Gap junctions allow for intercellular communication between adjacent connecting cells. GJs play a major role in the life cycle of cells; they are involved in tissue homeostasis and proliferation as well other aspects of the cell cycle [28-30]. In cancer cells, there is a significant change of Cx43 localization at the plasma membrane and the cytoplasmic membrane such as the golgi apparatus and the endolysosomal system [18,24]. PQ1, does not cause an increase in Cx43 expression, it causes a shift in the isoform expression causing Cx43 to once again be localized to the plasma membrane and to form functional gap junctions (Figures 4B and C and 5).

The regulation of gap junctions is controlled by phosphorylation of specific sites (mostly serine sites) on the carboxy-terminal tail region of the connexins [31]. Previously, Akt and MAPK have demonstrated to modulate Cx43 phosphorylation and subsequently increase gap junction activity. Active Akt has been found to stabilize gap junctions and active p44/42 MAPK has been shown to increase GJIC [12,13]. This study was also determined whether the increase of gap junction activity by PQ1 was due to the activation of Akt and MAPK. Interestingly, PQ1 was shown to cause an increase in activated Akt and p44/42 MAPK (Figures 6 and 7). This suggests the possibility that PQ1’s ability to increase GJIC is through activation of Akt and MAPK. PQ1’s ability to increase GJIC by kinase activity was tested using kinase inhibitors (Figure 8A and B). Results show that the increase in GJIC by PQ1 is at least in part due to kinase activity (Figure 8A and B).

## Conclusions

We have shown that GJIC can be restored via overexpression of Cx43 and by small molecule PQ1. PQ1 was shown to cause an increase in GJIC by changing the isoform expression of Cx43. Since PQ1 was designed using the structure of the c-terminus of Cx43 a potential mechanism may by direct binding to the c-terminus of Cx43. However this study has provided evidence of PQ1’s ability to cause an activation of Akt and p44/42 MAPK and subsequently increase GJIC in SW480 cells. Further studies are needed to elucidate the direct impact of PQ1 on specific site of phosphorylation of Cx43. Overall, the initial data provide insight into PQ1 being a mediator of kinase activity and via this activity cause an increase in GJIC of colorectal cancer cells. These data also conclude that PQ1 is a GJ enhancer in the SW480 colorectal cancer cells. This leads to the possibility of PQ1 being able to enhance the effects of current chemotherapeutic drugs by way of gap junctions.

## Abbreviations

Cx43: Connexin 43; GJIC: Gap junction intercellular communication; SL/DT: Scrape load/dye transfer; PQ1: 6-Methoxy-8[(3-aminopropyl) amino]-4methyl-5-(3-trifluoromethyl-phenloxy)quinoline; TPA: 12-O-Tetradecanoylphorbol-13-Acetate; Akt: Protein Kinase B; MAPK: Mitogen activated protein kinase.

## Competing interests

The authors declare that they have no competing interest.

## Authors’ contributions

KB designed and performed experiments as well as wrote the manuscript. TAN supervised and directed the studies as well as editing of the manuscript. Both authors read and approved the final manuscript.

## Pre-publication history

The pre-publication history for this paper can be accessed here:

http://www.biomedcentral.com/1471-2407/14/502/prepub
